# Insights Into the Ecology of a Widespread but Poorly Known Aerial Insectivore and a Theoretical Basis for Range Expansion Following Repeated Vagrancy Events

**DOI:** 10.1002/ece3.70576

**Published:** 2024-11-14

**Authors:** José R. Ramírez‐Garofalo

**Affiliations:** ^1^ Department of Ecology, Evolution, and Natural Resources Rutgers University New Brunswick New Jersey USA

**Keywords:** long‐distance dispersal, nesting ecology, northern rough‐winged swallow, range expansion, *Stelgidopteryx serripennis*, vagrancy

## Abstract

Over the course of the last two centuries, Northern Rough‐winged Swallows ( *Stelgidopteryx serripennis* ) have expanded their range across the North American continent but have remained a relatively poorly known species. In this paper, I discuss two aspects of their nature history that has received little attention. First, I document an instance of Northern Rough‐winged Swallows digging their own burrow, which was for the better part of the last century considered a behavior that was either lost or never occurred in the first place. Second, I review the natural history literature to document qualitative patterns evident in their expansion over the last two centuries. In doing so, I define three potentially useful concepts that can he applied to understand species' range shifts: an *Expansion Chronology*, which is a spatiotemporal map of a range shift; the concept of *Vagrancy‐induced Range Expansion*, where a species undergoes repeated movements outside of their typical geographic range (defined as vagrancy events), leading to the regular occurrence in a new region (e.g., regular overwintering or nonbreeding occurrences); and the concept of *Vagrancy‐induced Long‐distance Dispersal,* which is specifically expansion of a species' breeding range following repeated vagrancy events.

## Graphical abstract

Northern Rough‐winged Swallows are a widespread but poorly known aerial insectivore. Historically thought to only use the cavities of other species to nest, this paper confirms that they do in fact have the ability to excavate their own nesting burrows. Furthermore, this paper delves into the idea that the spread of urban areas across North America during the Gilded Age contributed to their range expansion, and the reduced need to dig their own burrows. Additionally, using the dispersal literature, the paper defines the pattern of expansion displayed by Northern Rough‐winged Swallows as Vagrancy‐induced Range Expansion.

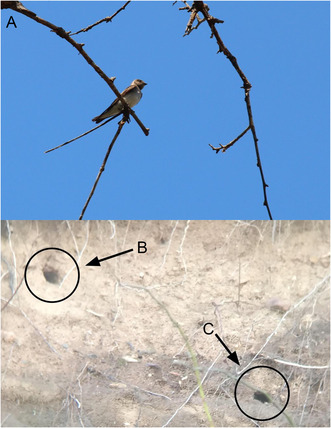

## 1 Introduction

Secondary cavity nesting species are species that nest in cavities that were already constructed by another species. These cavities include anthropogenic structures like nest boxes and drainpipes. Oftentimes, the exploitation of these structures allows these species to proliferate in urban and suburban settings, aiding them competitively (Guo et al. 2022), as well as in processes like range expansion when suitable habitat is also expanding (Uhrin et al. 2015).

Additionally, some species are widespread, common, and may be undergoing range expansion, but are poorly known to science. Emblematic of this is the Northern Rough‐winged Swallow ( *Stelgidopteryx serripennis* ). Northern Rough‐winged Swallows are neotropical migratory birds that breed throughout the North American continent. Despite their wide range and large population size, many aspects of their ecology are unknown (DeJong 1996) and almost everything that is known about their behavior and breeding habits are the result of one long‐term study conducted in Michigan, USA by Lunk (1962). While some recent work has examined molt and migration strategies in this species (Rohwer, Hobson, and Yang 2011), they have otherwise only been subject to occasional observational notes and little study.

One aspect of the life history of the Northern Rough‐winged Swallow that has been particularly contentious over the last century is their status as either a secondary cavity nester or a primary cavity excavator. Although they are widely accepted to be secondary cavity nesters, there is a lack of agreement in the historical literature on their ability to excavate their own nesting cavities. For example, Weydemeyer (1933) described Northern Rough‐winged Swallows as digging burrows in clay, sand, and gravel banks in northwestern Montana. However, in their survey of Ontario, Canada's breeding birds, Peck and James (1987) stated that Northern Rough‐winged Swallows sometimes dig their own burrows, but mainly use other available cavities. In Sage and Bishop's (1913) monograph on the birds of Connecticut, they stated that the species nests in burrows “usually dug by the birds themselves,” but acknowledged their propensity to also use other species' burrows and anthropogenic settings. Despite not finding any evidence of his own, Lunk (1962) remarked that Northern Rough‐winged Swallows probably did dig their own burrows, but only rarely.

Adding an additional dimension to the lack of study, and as a potential complement to the disagreement over their nesting ecology, Northern Rough‐winged Swallows have undergone a substantial multidirectional range expansion over the course of the last two centuries (DeJong 1996). However, none of the published literature has addressed the mechanism(s) behind this expansion. While expert observers like Bull (1985) and DeJong (1996) pointed out that increases in observer knowledge of bird identification may have contributed to the idea that this species has expanded their range, it is clear that they have in fact expanded their range substantially and continue to do so in multiple directions (Falardeau 2019).

In this paper, I describe an instance of a pair of Northern Rough‐winged Swallows digging their own burrows in a clay bluff in New York City, New York, USA during a long‐term study on coloniality in swallows. This confirms historical accounts of the species digging their own burrows, and Lunk's (1962) assumption that Northern Rough‐winged Swallows do sometimes dig their own burrows. Additionally, I discuss their range expansion within the context of expanding urbanization, which may have allowed this species to become more reliant on anthropogenic structures.

Using the range expansion of Northern Rough‐winged Swallows as a case study, I discuss the pattern evident in the natural history literature that points to Northern Rough‐winged Swallows making repeated movements outside of their geographic range (defined as vagrancy; Dufour et al. 2024), followed by settlement in new, unoccupied areas, which in turn has driven the rapidity of their expansion (see Kot, Lewis, and van den Driessche 1996). This pioneering behavior is evident across taxa and biological realms (Forconi et al. 2014; Davis and Watson 2018; Lees and Gilroy 2021; Dufour et al. 2024) and has recently become a far more important consideration in conservation and resource management decisions (Scheffers and Pecl 2019; Davis and Watson 2018). define some tools and concepts that may be useful in furthering our understanding of range expansions.

## 2 Methods

### 2.1 Field Methods

The observations reported here took place between 1 April and 15 August 2017 as part of an ongoing region‐wide study on swallow coloniality and persistence in urban areas (Ramírez‐Garofalo et al. 2021). During the course of this study, suitable colony locations for Bank and Northern Rough‐winged Swallow were located by searching all publicly accessible, coastal lands in the five boroughs of New York City (Brooklyn, Queens, Manhattan, The Bronx, and Staten Island) and surrounding New Jersey counties (Monmouth, Middlesex, Union, Essex, Hudson, and Bergen counties). Suitable natural locations for Bank Swallows—and by proxy Northern Rough‐winged Swallows—are usually clay or sand embankments, with slopes of near‐90° verticality (Ramírez‐Garofalo et al. 2021). When suitable sites were located, they were monitored on a daily basis by a team of researchers during the breeding season to determine if colonies would establish, and if they did so, the dynamics of their growth patterns, as well as observing predation events, with the goal of creating a management plan for bank‐nesting specialist species, some of which are imperiled in the region due to erosion mitigation measures and sea‐level rise.

The observations reported in this paper took place at Fort Wadsworth, Gateway National Recreation Area in Staten Island, New York, USA. The area is an active military installation that is coadministered by the US National Park Service as part of Gateway National Recreation Area—an urban national park established in 1972. Although the US Coast Guard and US Army Reserve are active within Fort Wadsworth, there are several historical, unoccupied military installations on‐site, including coastal defense batteries, forts, and escarpments, often made of limestone and dug into bluffs, forming a unique site where historical preservation and ecological conservation coincide.

### 2.2 Constructing an Expansion Chronology for the Northern Rough‐Winged Swallow

Documenting spatiotemporal patterns in species' range shifts can help cue us into potentially subtle but important qualitative aspects this process (Lenoir and Svenning 2015). Here, I map the expansion of Northern Rough‐winged Swallows in North America by reviewing the natural history literature pertinent to northeastern North America. This came in the form of various state, providential, and regional‐level ornithological texts, and the journal of Ornithological record, *North American Birds* (and its predecessor journals *American Birds* and *Audubon Field‐Notes*). This allowed me to gather both spatiotemporal data on their range expansion, as well as direct opinions from period‐specific Ornithologists on their regional status. I also consulted the Web of Science database, for which I seared “All Fields” using the queries “Northern Rough‐winged Swallow” OR “Rough‐winged Swallow.” The addition of “Rough‐winged Swallow” stemmed from the split of the species into Northern Rough‐winged Swallow and Southern Rough‐winged Swallow in 1983 (see Stiles 1981; American Ornithologists Union 1983). The results of this search are in Data S1. Since some of the natural history literature is not present in the Web of Science database, I queried the Searchable Ornithological Research Archive (SORA) hosted by the University of New Mexico, which often contains Ornithological literature from journals not indexed in the Web of Science (e.g., some state/regional journals; Table S1). In addition, I searched each of the references presented in DeJong (1996), which remains the most comprehensive compilation of information on this species. I refer to the mapped‐out results of this review as an Expansion Chronology (see Figure 2).

## 3 Results

### 3.1 Field Observations of Burrow Excavation

**FIGURE 1:**
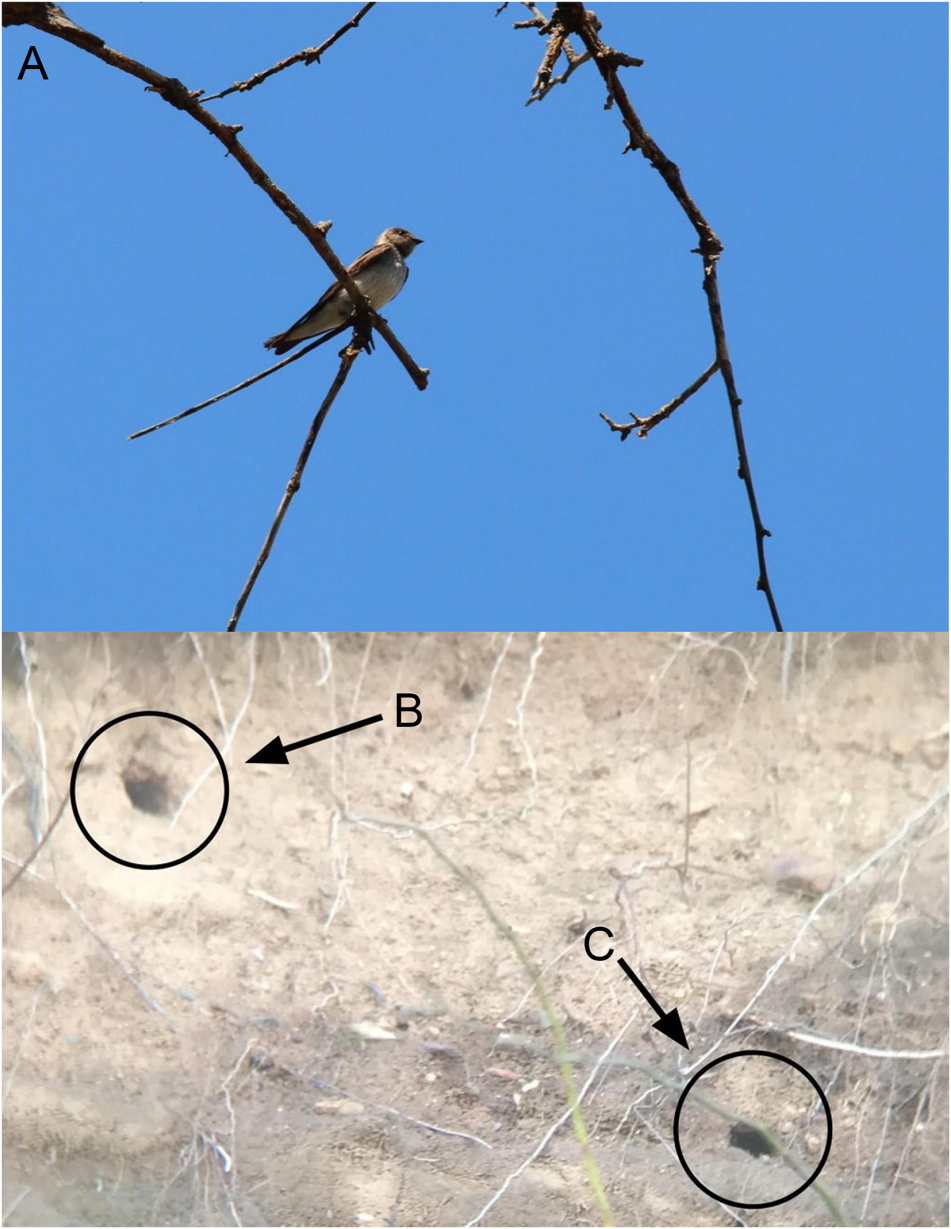
In (A), an adult Northern Rough‐winged Swallow ( *Stelgidopteryx serripennis* ) is perched on a tree directly in front of its nesting site. Northern Rough‐winged Swallow burrows dug into a clay embankment in Staten Island, New York, USA. The active burrow excavated by the pair of swallows (and burrow that was used through the breeding season) is indicated by (B), while the inactive burrow is indicated by (C).

On 3 May 2017, I visited a bluff with a recently exposed (< 6 months) clay deposit under the Verrazano‐Narrows Bridge in Staten Island, NY. The deposit had been uncovered during a federal hurricane recovery project to shore up eroding cliff faces at Fort Wadsworth, Gateway National Recreation Area following devastation by the landfall of Hurricane Sandy in late‐October 2012. Although there were no swallow burrows present, there was a single occupied Belted Kingfisher ( *Megaceryle alcyon* ) burrow that had already been active for over 1 month. That day, at 1045 h, I observed a pair of Northern Rough‐winged Swallows (Figure 1A) foraging and displaying under the Verrazano‐Narrows Bridge in the vicinity of the bluffs. Between 4 May and 12 May, I made daily site visits, but did not detect any Bank Swallows, nor any burrows aside from the burrow that was already occupied by the Belted Kingfishers. On 13 May 2017, I returned to the site at 1000 h and found a partially excavated burrow approximately 1 meter above the Belted Kingfisher burrow (Figure 1C). The burrow was small and round, unlike that of a Belted Kingfisher, and was being actively visited by two Northern Rough‐winged Swallows. I returned the next day, 14 May, at 1000 h and the burrow appeared to be completely dug, showing typical signs of recent occupation (i.e., entrance with fresh claw marks, lack of vegetation hanging directly over the entrance hole). However, upon closer approach no birds left as would be typical of an occupied burrow. The next morning, 15 May, I once again returned to the site, this time finding another partially dug burrow approximately one‐half meter above the burrow I had found on 13 May (Figure 1B). One Northern Rough‐winged Swallow was inside the burrow kicking sand out and quickly exited upon my arrival. Both members of the pair of swallows flew around the holes for several minutes but would not enter them. I retreated about 200 ft and observed one of the swallows enter the burrow and resume kicking sand out. I returned to the site on 16 May, observing both swallows exit the burrow when I stood on the beach in front of the bluff. The second burrow, which at this point seemed to have been complete, was also small and round, unlike that of a Belted Kingfisher. The pair remained in the area, occupying the burrow until 9 July, but did not successfully fledge young.

### 3.2 Expansion Chronology

**FIGURE 2:**
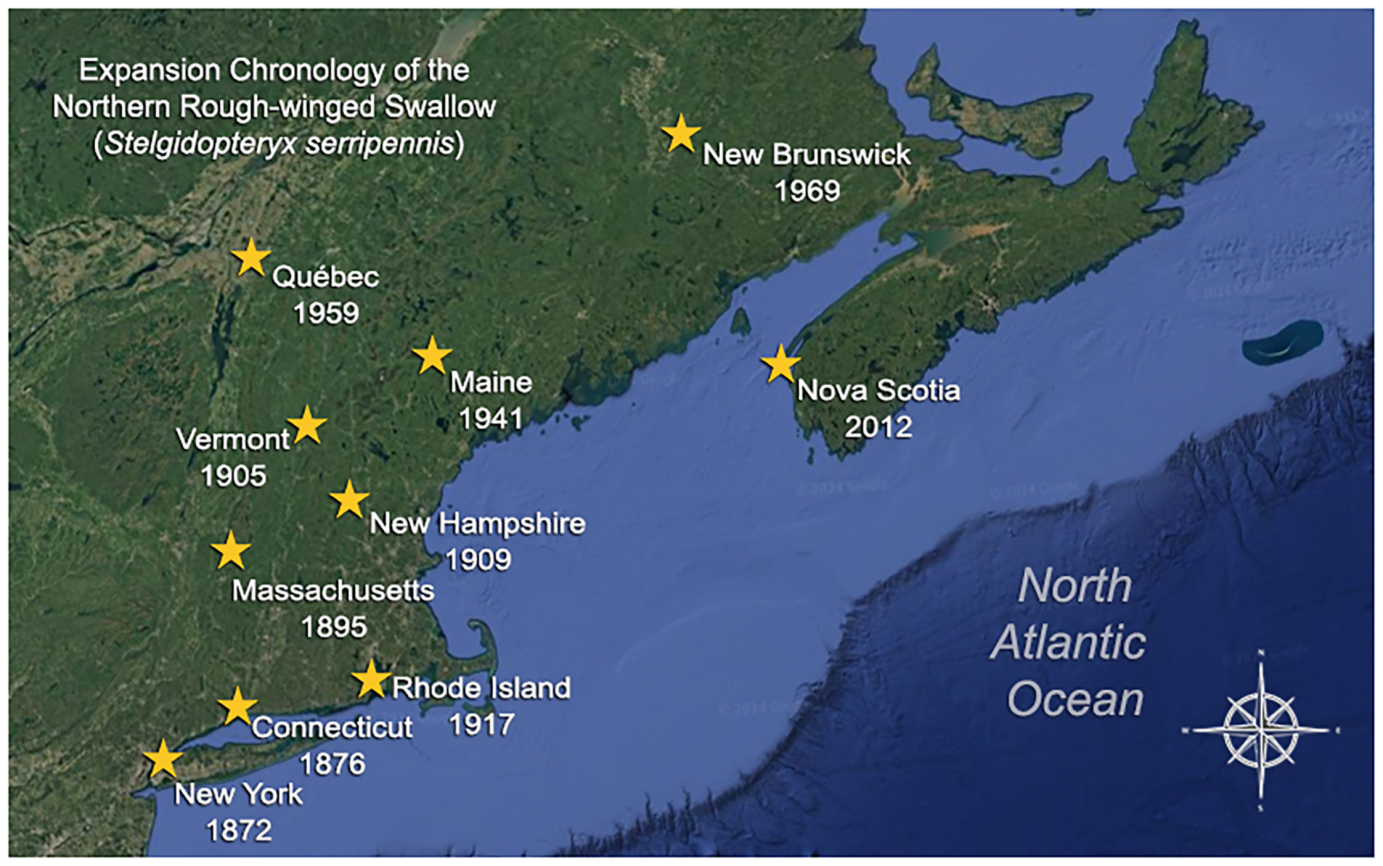
Chronology of the expansion of Northern Rough‐winged Swallows in northeastern North America. Stars indicate the approximate locations of the first nesting records, as recorded in the natural history literature.

During the 19th and 20th centuries, Northern Rough‐winged Swallows dramatically expanded their range, particularly in northeastern North America and by the latter‐half of the 20th century, from the south and west (DeJong 1996; see Figure 2).

Focusing on the northeast, which has had particularly good observer coverage over the last two centuries (Veit and Petersen 1993), we see that first breeding records were recorded for New York in 1872 (Bull 1985), Connecticut in 1876 (Stannis 1879), Rhode Island in 1917 (Ferren 2024), Massachusetts in 1895 (Veit and Petersen 1993), Vermont in 1905 (Norse 1985), New Hampshire in 1909 (May 1930), Maine in 1941 (Vickery et al. 2020), Québec in 1959 (Falardeau 2019), and New Brunswick in 1969 (Squires 1976). They were found breeding for the first time in Nova Scotia in 2012, but they remain rare in the province (McLaren 2012) and throughout the Canadian Maritimes (Bredin 2015).

## 4 Discussion

As made clear by the observations presented here, Northern Rough‐winged Swallows at least occasionally act as primary cavity excavators. This confirms Lunk's (1962) assumption that Northern Rough‐winged Swallows may excavate their own burrows on rare occasion and highlights the importance of natural history in an era when it is declining in favor of larger‐scale ecological study (Anderson 2017). This observation is the first explicit description in the literature of burrows being excavated solely by Northern Rough‐winged Swallows. Although they are known to nest in natural settings that are not necessarily burrows dug by other species like (DeJong 1996), this is the first description of a burrow that is entirely newly dug by the swallows themselves. Therefore, we should refer to Northern Rough‐winged Swallows as weak primary cavity nesters (*sensu* McClelland, Frissell, and Fischer 1979), which are species that have the ability to dig their own cavities, but usually do not do so.

Species range shifts occur as a result of a number of biotic and abiotic factors (Chen et al. 2011; Lenoir and Svenning 2015). Over the last century, range shifts have occurred at an increased rate, owing mainly to anthropogenic alterations to the biosphere (Loarie et al. 2009; Urban 2015). These changes can occur at a variety of different speeds and are often driven by changes in temperature, precipitation, and land‐use (Loarie et al. 2009; Chen et al. 2011; Urban 2015). In many cases, anthropogenic land‐use change can directly facilitate range expansions, particularly for species considered to be synanthropic (e.g., Uhrin et al. 2015). In the case of Northern Rough‐winged Swallows, land‐use changes—namely urbanization—provided a massive increase in nesting opportunities over the course of their north and eastward expansion.

Previously, Bull (1985) and DeJong (1996) both hypothesized that their expansion was possibly an artifact of increased observer awareness of their identification. However, I posit that the species' willingness to nest within anthropogenic structures like drainpipes facilitated their expansion. Cavity nesters have been found to benefit from urbanization (Tomasevic and Marzluff 2018), and the expansion of Northern Rough‐winged Swallows began at a time when the northeastern United States was going through a period of widespread urbanization and land‐use change. Specifically, during and immediately following the Gilded Age (approximately 1869–1901), the northeastern United States saw an unprecedented and rapid increase in urbanization (Schlesinger 1933), and first breeding records for northeastern states aligned with the regions' major industrialization period (Temin 1999). Highlighting their potential association with urbanization there were no records of this species prior to the widespread urbanization of the northeastern United States, with most “first‐records” for individual states coming just prior to first breeding records (e.g., New York, Massachusetts, and Vermont; Bull 1985; Norse 1985; Veit and Petersen 1993).

### 4.1 The Concept of Vagrancy‐Induced Range Expansion and Long‐Distance Dispersal

Adding to the interesting nature of their range expansion is the dispersal strategy that they employ to do so. As discussed above, Northern Rough‐winged Swallows occurred first as vagrants prior to first breeding records in nearly every instance recorded in the literature (DeJong 1996). Rather than short‐distance diffusion‐like movements that caused their range boundary to creep slowly northward (Hengeveld 1989; Kot, Lewis, and van den Driessche 1996; Lewis, Petrovskii, and Potts 2016), Northern Rough‐winged Swallows underwent long‐distance, jump‐like movements outside of their geographic range without initially settling. Although upon establishment this resembles long‐distance dispersal *sensu stricto* (Jordano 2017), where individuals undergo extremely long‐distance movements leading to gene flow (Ronce 2007; Clobert et al. 2009), we see here a narrower form of long‐distance dispersal. Northern Rough‐winged Swallows specifically moved *outside* of their range, and after some period of time where they were vagrants, they established and continued to expand. Considering the mounting evidence that this specific phenomenon of establishment following repeated vagrancy events is prevalent across many taxa (Dufour et al. 2024), we can and should give it a specific label as to differentiate it from typical long‐distance dispersal. As such, I will refer to this as *Vagrancy‐induced Range Expansion* and define it as “range expansion that follows initial, repeated occurrences by vagrant organisms leading up to their regular occurrence in an area previously not recorded to be part of their geographic range.” The idea of this being separate from what we would normally consider long‐distance dispersal is important, as we are seeing that vagrancy is leading to general range expansion across a number of taxa (Fogarty et al. 2017; Dufour et al. 2024), as well as the emergence of new migration routes in birds (Dufour et al. 2021, 2022, 2024; Lees and Gilroy 2021; Bozó and Csörgő 2024).

When this process is narrowed down further to breeding range expansion, we can consider this *Vagrancy‐induced Long‐distance Dispersal*, which I will define here as “breeding‐range expansion following repeated vagrancy events, leading specifically to gene flow from a species' typical geographic range into a region that was not previously known to be occupied by the species.” As in Kot, Lewis, and van den Driessche (1996), these types of range expansions and long‐distance dispersal events should cause expanding ranges to be faster than what would normally occur under a scenario where expansion occurs because of short‐distance dispersal. These jumps can then lead to more diffusion‐like range expansion from newly established populations, such as what is evident for Northern Rough‐winged Swallows in areas between successive jumps. For instance, long‐distance dispersal between Connecticut and Massachusetts occurred between 1876 and 1895, which was followed by slower movement eastward during which repeated vagrancy events occurred but were much closer to the breeding range (Ferren 2024). This resulted in eventual breeding in Rhode Island in 1917, about a decade later than other northern states like Vermont and New Hampshire. This pattern of long‐distance movement, followed by diffusion‐like movements is often considered in the invasion ecology literature (called Stratified Diffusion; Hengeveld 1989; Kot, Lewis, and van den Driessche 1996; Lewis, Petrovskii, and Potts 2016), but here we see a clear application in the natural movement of native species.

Overall, these concepts, taken together with an Expansion Chronology, can help clarify patterns of range shifts and/or range expansion. For species in areas with larger volumes of data over a longer period of time, this is particularly true. For those occurring in areas with lower observer coverage, or those that are only recently occurring following vagrancy or long‐distance dispersal events from other regions, an Expansion Chronology can be a first step for natural resource managers that are making decisions on whether to conserve a particular range‐shifting species (e.g., Scheffers and Pecl 2019).

### 4.2 Future Research Needs for Northern Rough‐Winged Swallows

Northern Rough‐winged Swallows require additional study beyond occasional and incidental observations. With a focus toward conservation under current climate and land‐use conditions, determining the reasoning behind some of the other ambiguous aspects of their ecology is deserved. For example, there is a sizable, disjoint wintering population in the mid‐Atlantic United States (located in Pennsylvania), which is over one thousand kilometers north of the closest part of their winter range, located in southern Florida. Understanding the process by which they have expanded to this more northerly location is important and potentially applicable to other species that display disjoint, nonbreeding movements that lead to expansion of the nonbreeding range. As discussed in this paper, there are range shifts that occur on a disjoint basis via long‐distance dispersal and what I have defined as a vagrancy‐induced range expansion, which need to be considered as we study the global redistribution of species. In the case of Northern Rough‐winged Swallows, this disjoint wintering population was established following a period of increased winter vagrancy by this species, as documented in the southern United States by Johnson (2005), and evident in records posted to the eBird databased (Sullivan et al. 2014).

## Author Contributions

**José R. Ramírez‐Garofalo:** conceptualization (lead), investigation (lead), methodology (lead), project administration (lead).

## Conflicts of Interest

The author declares no conflicts of interest.

## Supporting information


Data S1.

Table S1.

## Data Availability

All data generated during the study is contained within the article and in Supporting Information.
