# Supplementary material for: Insights Into the Ecology of a Widespread but Poorly Known Aerial Insectivore and a Theoretical Basis for Range Expansion Following Repeated Vagrancy Events
Source: Ecol Evol. 2024 Nov 14;14(11):e70576. doi: 10.1002/ece3.70576 (PMC11563706; doi:10.1002/ece3.70576)
Supplement: Supplementary file 1 — Data S1. [file ECE3-14-e70576-s001.docx]

**Supplementary Material 2**

**References consulted from De Jong (1996):**

Able, K. P. (1972). Fall migration in coastal Louisiana and the evolution of migration patterns in the Gulf region. Wilson Bulletin 84:231-242.

- Adamus, P. R. (1987). Atlas of Breeding Birds in Maine, 1978–1983. Maine Department of Inland Fisheries and Wildlife, Augusta, ME, USA.
- American Ornithologists' Union (1983). Check-list of North American Birds. 6th edition. American Ornithologists' Union, Washington, DC, USA. <https://www.biodiversitylibrary.org/page/34735248>
- Audubon, J. J. (1838). Ornithological Biography. Volume 4. Adam & Charles Black, Edinburgh, Scotland.

Beal, F. E. L. (1918). Food habits of the swallows, a family of valuable native birds. Bulletin of the U.S. Department of Agriculture 619: 1–28.

- Beecher, M. D., M. B. Medvin, P. K. Stoddard, and P. Loesche (1986). Acoustic adaptations for parent-offspring recognition in swallows. Journal of Experimental Biology 45: 179–183.
- Behle, W. H. (1985). Utah Birds: Geographic Distribution and Systematics. Occasional Publication Number 5. Utah Museum Natural History, Salt Lake City, UT, USA.
- Bent, A. C. (1942). Life histories of North American flycatchers, larks, swallows, and their allies. U.S. National Museum Bulletin 179, Smithsonian Institution, Washington, DC, USA.
- Best, L. B. (1977a). Bullsnake preys on Rough-winged Swallow nest. Condor 79:509.
- Blake, C. H. (1947a). Swallow notes. Bull. Mass. Aud. Soc. 31:239.
- Blake, C. H. (1948b). The flight of swallows. Auk 65: 54–62.
- Blake, C. H. (1953a). Notes on the Rough-winged Swallow. Bird-Banding 24:107-108.
- Blake, F. G. (1907). The nesting of *Stelgidopteryx serripennis* in Norwich, VT. Auk 24:103-104.
- Bohlen, H. D. (1989). The Birds of Illinois. Indiana University Press, Bloomington, IN, USA.
- Bond, J. (1974). Birds of the West Indies. 4th ed. London: Collins.
- Brewer, R. G., A. McPeak, and R. J. Adams Jr. (1991). The Atlas of Breeding Birds of Michigan. Michigan State University Press, East Lansing, MI, USA.
- Brodkorb, P. (1942). Notes on some races of the Rough-winged Swallow. Condor 44:214-217.
- Brown, C. R., and J. L. Hoogland (1986). Risk in mobbing for solitary and colonial swallows. Animal Behaviour 34: 1319–1323.
- Bull, J. (1974). Birds of New York State. Doubleday Natural History Press, Garden City, NY, USA.
- Burleigh, T. D. (1944b). The bird life of the Gulf Coast region of Mississippi. Occas. Pap. Mus. Zool. La. State Univ. 20:329-490.
- Burleigh, T. D. (1972). Birds of Idaho. Caxton Printers, Caldwell, ID, USA.
- Burtt, Jr., E. H., E. J. Bitterbaum and J. P. Hailman. (1988). Head-scratching method in swallows depends on behavioral context. Wilson Bulletin 100:679.
- Cadman, M. D., P. F. J. Eagles, and F. M. Helleiner (1987). Atlas of Breeding Birds of Ontario. University of Waterloo Press, Waterloo, Canada.
- Campbell, R. W., N. K. Dawe, I. McTaggart-Cowan, J. M. Cooper, G. W. Kaiser, M. C. E. McNall, and G. E. J. Smith (1997). The Birds of British Columbia. Volume 3. Passerines: Flycatchers Through Vireos. University of British Columbia Press, Vancouver, Canada.
- Cannings, R. A., R. J. Cannings, and S. G. Cannings (1987). Birds of the Okanagan Valley, British Columbia. Royal British Columbia Museum, Victoria, BC, Canada.
- Cyr, A., and J. Larivee (1995). Atlas Saisonnier des Oiseaux du Quebec. Les Presses de l'Universidad de Sherbrooke et la Societe de Loisir Ornithologique de l'Estrie, Inc., Sherbrooke, Canada.
- Davie, O. (1898). Nests and Eggs of North American Birds. 5th edition. David McKay, Philadelphia, Pennsylvania, USA.
- Dickey, D. R., and A. J. van Rossem (1938). The birds of El Salvador. Field Museum of Natural History Zoological Series, Chicago, Illinois, USA. <https://www.biodiversitylibrary.org/page/2779539>
- Dingle, E. (1942). "Rough-winged Swallow." In Life histories of North American flycatchers, larks, swallows, and their allies., edited by A. C. Bent, 424-433. U.S. Natl. Mus. Bull. 179.
- Droege, S., and J. R. Sauer (1990). North America breeding bird survey annual summary, 1989. U.S. Fish and Wildlife Service Biological Report 90 (8).
- Dunning, J. B., Jr. (1993). CRC Handbook of Avian Body Masses. CRC Press, Boca Raton, FL, USA.
- Dwight, J., Jr. (1900). The sequence of plumages and moults of the passerine birds of New York. Annals of the New York Academy of Sciences 13:73–360. <https://www.biodiversitylibrary.org/bibliography/23225>
- Erskine, A. J. (1979). Man's influence on potential nesting sites and populations of swallows in Canada. Canadian Field-Naturalist 93:371-377.
- Erskine, A. J. (1992). Atlas of Breeding Birds of the Maritime Provinces. Nova Scotia Museum, Halifax, Canada.
- Eynon, A. E. (1936). Rough-winged Swallow breeding in Rhode Island. Auk 53:83-84.
- Faaborg, J. R., and J. W. Terborgh (1980). Patterns of migration in the West Indies. In Migrant Birds in the Neotropics: Ecological, Behavior, Distribution and Conservation (A. Keast and E. S. Morton, Editors). Smithsonian Instution Press, Washington, DC, USA.
- Foss, C. R. (1994). Atlas of Breeding Birds in New Hampshire. Arcadia Publishing, Mount Pleasant, SC, USA.
- Friedmann, H., L. Griscom, and R. Moore (1950). Distributional checklist of the birds of Mexico, Part I. Pacific Coast Avifauna 29.
- Gillespie, J. A. (1934). The homing instinct in the Rough-winged Swallow. Bird-Banding 5:43-44.
- Gilligan, J. D., M. Rogers, A. Smith, and A. Contreras (1994). Birds of Oregon: Status and Distribution. Cinclus Publications, McMinnville, OR, USA.
- Godfrey, W. E. (1986). The Birds of Canada. Revised Edition. National Museums of Canada, Ottawa, Canada.
- Graber, R. R., J. W. Graber, and E. L. Kirk (1972). Illinois birds: Hirundinidae. Illinois Natural History Survey Biological Notes 80.
- Haggerty, T. (1981). Rat snake preys on nestlings of Rough-winged Swallow and Common Grackle. Chat 45:77.
- Hanna, W. C. (1924). Weights of about three thousand eggs. Condor 26:146–153.
- Harrison, C. A. (1984). A Field Guide to the Nests, Eggs, and Nestlings of North American Birds. Stephen Greene Press, Brattleboro, VT, USA.
- Hartman, F. A. (1961). Locomotor mechanisms in birds. Smithsonian Miscellaneous Collection 143:1–99. <https://repository.si.edu/bitstream/handle/10088/22973/SMC_143_Hartman_1961_1_1-91.pdf?sequence=1>
- Headstrom, R. (1970). A complete field guide to nests in the United States. New York: Ives Washburn Inc.
- Hespenheide, H. A. (1975). Selective predation by two swifts and a swallow in Central America. Ibis 117:82-99.
- Hess, I. E. (1910). One hundred breeding birds of an Illinois ten-mile radius. Auk 27:19-32.
- Hill, J. R. (1988d). Nest-depth preference in pipe-nesting Northern Rough-winged Swallows. Journal of Field Ornithology 59:334-336.
- Howell, S. N. G. (1989a). Additional information on the birds of the Campeche Bank, Mexico. Journal of Field Ornithology 60:504-509.
- Howell, S. N. G., and S. Webb (1995). A Guide to the Birds of Mexico and Northern Central America. Oxford University Press, New York, NY, USA.
- Husmann, K. H. (1981). Rough-winged Swallows nest in building. S. D. Bird Notes 33:59.
- Janssen, R. B. (1987). Birds in Minnesota. University of Minnesota Press, Minneapolis, MN, USA.
- Jewett, S. G., W. P. Taylor, and J. W. Aldrich (1953). Birds of Washington State. University of Washington Press, Seattle, WA, USA.
- Johnson, N. K. (1994c). Old-school taxonomy versus modern biosystematics: species-level decision in *Stelgidopteryx* and *Empidonax*. Auk 111:773-780.
- Jones, H.L. (2004). Birds of Belize. Christopher Helm, London.
- Kessel, B., and D. Gibson (1978). Status and distribution of Alaska birds. Studies in Avian Biology 1:1–100. <http://www.arlis.org/docs/vol1/4749617.pdf>
- Kingery, H. E. and U. C. Kingery. (1995a). Gopher snake as predator at Long-billed Curlew and Rough-winged Swallow nests. Colo. Field Ornithol. 29:18-19.
- Lethaby, N. (1996). Identification of Tree, Northern Rough-winged, and Bank Swallows. Birding 28:111-116.
- Lunk, W. A. (1962). The Rough-winged Swallow: a study based on its breeding biology in Michigan. Publications of the Nuttall Ornithological Club 4.
- Macoun, J., and J. M. Macoun (1909). Catalogue of Canadian Birds. Government Printing Bureau, Ottawa, Ontario, Canada.
- Michael, Jr., J. H. (1992). Intertidal nest of Northern Rough-winged Swallow. Wash. Birds 2:23-24.
- Mumford, R. E., and C. E. Keller (1984). The Birds of Indiana. Indiana University Press, Bloomington, Indiana, USA.
- Nickell, W. P. (1949). A large nest of the Rough-winged Swallow. Wilson Bulletin 61:188-189.
- Niethammer, K. R., R. B. Atkinson, T. S. Baskett and F. B. Samson. (1985). Metals in riparian wildlife of the lead mining district of southeastern Missouri. Archives of Environmental Contamination and Toxicology 14:213-223.
- Ouellet, H. (1970a). Changes in the bird fauna of the Montreal region, Canada. Canadian Field-Naturalist 84:27-34.
- Peck, G. K., and R. D. James (1987). Breeding Birds of Ontario: Nidiology and Distribution. Volume 2: Passerines. Miscellaneous Publications of the Royal Ontario Museum, Toronto, Canada.
- Peterjohn, B. G. (1989). The Birds of Ohio. Indiana University Press, Bloomington, IN, USA.
- Peters, H. S. (1936). A list of external parasites from birds of the eastern part of the United States. Bird-Banding 7:9–27.
- Phillips, A. R. (1986). The Known Birds of North and Middle America. Part I: Hirundinidae to Mimidae; Certhiidae. Denver Museum of Natural History, Denver, CO, USA.
- Phillips, A. R. (1994c). *The known birds of North and Middle America* versus the current AOU list. Auk 111:770-773.
- Platania, S. P. and M. K. Clark. (1981). Rough-winged Swallow nesting in coastal North Carolina. Chat 45:100-102.
- Pyle, P. (1997). Identification Guide to North American Birds. Part I. Slate Creek Press, Bolinas, CA, USA.
- Raffaele, H. A. (1989). A guide to the birds of Puerto Rico and the Virgin Islands. Princeton University Press, Princeton, NJ, USA.
- Ricklefs, R. E. (1972). Latitudinal variation in breeding productivity of the Rough-winged Swallow. Auk 89:826-836.
- Ridgely, R. S., and J. Gwynne (1989). A Guide to the Birds of Panama, with Costa Rica, Nicaragua, and Honduras. 2nd edition. Princeton University Press, Princeton, New Jersey, USA.
- Ridgway, R. (1904). The Birds of North and Middle America. Part III. Bulletin of the United States National Museum 50. <https://www.biodiversitylibrary.org/item/32323#page/7/mode/1up>
- Robbins, C. S., D. Bystrack, and P. H. Geissler (1986). The Breeding Bird Survey: its first fifteen years, 1965–1979. U.S. Fish and Wildlife Service Resource Publication 157.
- Robbins, M. B., and D. A. Easterla (1992). Birds of Missouri: Their Distribution and Abundance. University of Missouri Press, Columbia, MO, USA.
- Robbins, S. D., Jr. (1991). Wisconsin Birdlife: Population and Distribution, Past and Present. University of Wisconsin Press, Madison, WI, USA.
- Robertson, Jr., W. B. and J. A. Kushlan. (1974). "The southern Florida avifauna." In Environments of south Florida: present and past. Miami Geol. Soc. Mem. 2:414-452.
- Robertson, W. B., Jr., and G. E. Woolfenden (1992). Florida Bird Species: an Annotated List. Florida Ornithological Society Special Publication 6, Florida Ornithological Society, Gainesville, FL, USA.
- Rosenberg, K. V., R. D. Ohmart, W. C. Hunter, and B. W. Anderson (1991). Birds of the Lower Colorado River Valley. University of Arizona Press, Tucson, AZ, USA.
- Rowley, J. S. (1966). Breeding records of birds of the Sierra Madre del Sur, Oaxaca, Mexico. Proceedings of the Western Foundation of Vertebrate Zoology 1(3):107–204.
- Sargent, H. B. (1893). Breeding of the Rough-winged Swallow at Shelter Island, New York. Auk 10:369.
- Saunders, A. A. (1951). A Guide to Bird Songs. Doubleday, New York, NY, USA.
- Screws, Jr., D. G. (1991). Unusual behavior in Northern Rough-winged Swallows. Oriole 56:44-45.
- Sealy, S. G. (1982a). Rough-winged Swallow scavenging adult midges. Wilson Bulletin 94:368-369.
- Semenchuk, G. P. (1992). The Atlas of Breeding Birds of Alberta. Federation of Alberta Naturalists, Edmonton, Canada.
- Sheldon, F. H., and D. W. Winkler (1993). Intergeneric phylogenetic relationships of swallows estimated by DNA-DNA hybridization. Auk 110: 798–824.
- Skutch, A. F. (1960b) Life histories of Central American birds. II. Pacific Coast Avifauna 34. <https://sora.unm.edu/sites/default/files/journals/pca/pca_034.pdf>
- Skutch, A. F. (1981) New studies of tropical American birds. Publications f the Nuttall Ornithological Club 19. <https://www.biodiversitylibrary.org/page/57272736>
- Small, A. (1994). California Birds: Their Status and Distribution. Ibis Publishing Company, Vista, CA, USA.
- Snyder, D. E. (1950a). Death of a Rough-winged Swallow, *Stelgidopteryx ruficollis*. Auk 67:390.
- Speirs, J. M. (1985). Birds of Ontario. Natural History/Natural Heritage Inc., Toronto, ON, Canada.
- Starrett, W. C. (1938). Highway casualties in central Illinois during 1937. Wilson Bulletin 50:193-196.
- Stedman, S. J. and D. J. Simbeck. (1988). Northern Rough-winged Swallows build nests in semi-trailers. Migrant 59:51-52.
- Stevenson, H. M., and B. H. Anderson (1994). The Birdlife of Florida. University Press of Florida, Gainesville, FL, USA.
- Stiles, F. G. (1981b). The taxonomy of Rough-winged Swallows (*Stelgidopteryx*; Hirundinidae) in southern Central America. Auk 98:282-293.
- Stiles, F. G., and A. F. Skutch (1989). A Guide to the Birds of Costa Rica. Cornell University Press, Ithaca, New York, USA.
- Turner, A., and C. Rose (1989). Swallows and Martins: An Identification Guide and Handbook. Houghton Mifflin Company, Boston, MA, USA.
- Van Fleet, W. (1876). Notes on the Rough-winged Swallow (*Hirundo serripennis*), in Pennsylvania. Bulletin of the Nuttall Ornithological Club 1:9-11.
- Veit, R. R., and W. R. Petersen (1993). The Birds of Massachusetts. Massachussetts Audubon Society, Lincoln, MA, USA.
- Voous, K. H. (1983). Bird of the Netherlands Antilles. Foundation for Scientific Research in Surinam and the Netherlands, Antilles, Utrecht, The Netherlands.
- Wauer, R. H. (1985). A Field Guide to Birds of the Big Bend. Texas Monthly Press, Austin, TX, USA.
- Webster, E. E. (1915). Rough-winged Swallows in New Hampshire. Bird-Lore 17:293.
- Wetmore, A. (1936). The number of contour feathers in Passeriform and related birds. Auk 53: 159–169.
- Wetmore, A. (1984). The birds of the Republic of Panama, Pt. 4. Passeriformes. Hirundinidae to Fringillidae. Completed by R. F. Pasquier and S. L. Olson. Smithson. Misc. Coll. 150.
- Weydemeyer, W. (1933). Nesting of the Rough-winged Swallow in Montana. Auk 50:362-363.
- Wilds, C. (1985b). Unraveling the mysteries of brown swallows. Birding 17:209-211.
- Wolinski, R. A. (1980). Rough-winged Swallow feeding of fly larvae. Wilson Bulletin 92:121-122.
  - Amos, E.J.R. (1991). A Guide to the Birds of Bermuda. Published privately, Warwick.
  - Arendt, W.J. (1992). Status of North American migrant landbirds in the Caribbean region: a summary. Pp. 143–171 in: Hagan & Johnston (1992).
  - Armstrong, R. H. (1983). A New, Expanded Guide to the Birds of Alaska. Alaska Northwest Publishing Company, Anchorage, AK, USA.
  - Babin, M.J. (2005). Geographic Variation and Speciation in Rough-winged Swallows (Aves: Hirundinidae: Stelgidopteryx). MSc thesis, Louisiana State University, Baton Rouge, Louisiana.
  - Baicich, P. J., and C. J. O. Harrison (1997). A Guide to the Nests, Eggs, and Nestlings of North American Birds. Second edition. Academic Press, San Diego, CA, USA.
  - Baicich, P.J. and Harrison, C.J.O. (1997). A Guide to the Nests, Eggs, and Nestlings of North American Birds. Academic Press, San Diego, California.
  - Beecher, M.D., Medvin, M.B., Stoddard, P.K. and Loesche, P. (1986). Acoustic adaptations for parent-offspring recognition in swallows. Journal of Experimental Biology. 45: 179–183.
  - Behle, W.H. (1985). Utah Birds: Geographic Distribution and Systematics. Utah Museum of Natural History, Salt Lake City, Utah.
  - Bent, A.C. (1942). Life Histories of North American Flycatchers, Larks, Swallows and their Allies. US National Museum Bulletin 179. Smithsonian Institution, Washington, DC, USA.
  - Best, L.B. (1977). Bullsnake preys on Rough-winged Swallow nest. Condor. 79(4): 509.
  - Binford, L. C. (1989). A Distributional Survey of the Birds of the Mexican State of Oaxaca. Ornithological Monographs 43. American Ornithologists’ Union. Washington, DC, USA.
  - Blake, C.H. (1953). Notes on the Rough-winged Swallow. Bird-Banding. 24: 107–108.
  - Blake, F.G. (1907). The nesting of Stelgidopterix serripennis in Norwich, VT. Auk. 24(1): 103–104.
  - Boesman, P. (2016). Notes on the vocalizations of Northern Rough-winged Swallow (*Stelgidopteryx serripennis*). HBW Alive Ornithological Note 239. In: Handbook of the Birds of the World Alive. Lynx Edicions, Barcelona. <https://doi.org/10.2173/bow-on.100239>
  - Brand, L.A., Stromberg, J.C. and Noon, B.R. (2010). Avian density and nest survival on the San Pedro River: importance of vegetation type and hydrologic regime. Journal of Wildlife Management. 74(4): 739–754. <https://doi.org/10.2193/2008-217>
  - Brodkorb, P. (1942). Notes on some races of the Rough-winged Swallow. Condor. 44(5): 214–217.
  - Brown, C.R. and Hoogland, J.L. (1986). Risk in mobbing for solitary and colonial swallows. Animal Behaviour. 34: 1319–1323.
  - Campbell, R.W., Dawe, N.K., McTaggart-Cowan, I., Cooper, J.M., Kaiser, G.W., McNall, M.C.E. and Smith, G.E.J. (1997). The Birds of British Columbia. Vol. 3. Flycatchers through Vireos. British Columbia Ministry of Environment, Lands and Parks and Environment Canada, Victoria & Delta, British Columbia.
  - Clapp, R.B. (1992). Northern Rough-winged Swallow building nest in semi-trailer. Raven 63: 72–73.
  - Contreras-Balderas, A. J. (1997). Resumen avifaunístico de Nuevo León, México. In The Era of Allan Phillips: A Festschrift (R. W. Dickerman, Editor), Horizon Communications, Albuquerque, NM, USA. pp. 35–44.
  - Cyr, A. and Larivée, J. eds. (1995). Atlas Saisonnier des Oiseaux du Québec. Presses de l’Université de Sherbrooke & Société Ornithologique de l’Estrie, Sherbrooke, Québec.
  - DeGraaf, R. M., and D. D. Rudis (1986). New England Wildlife: Habitat, Natural History and Distribution. USDA Forest Service General Technical Report NE-108.
  - DeGraaf, R. M., and J. H. Rappole (1995). Neotropical Migratory Birds: Natural History, Distribution, and Population Change. Cornell University Press, Ithaca, NY, USA.
  - DeGraaf, R.M. and Rappole, J.H. (1995). Neotropical Migratory Birds. Natural History, Distribution, and Population Change. Comstock Publishing Associates, Ithaca & London.
  - DeJong, M.J. (1996). Northern Rough-winged Swallow (Stelgidopteryx serripennis). No. 234 in: Poole, A.F. & Gill, F.B. eds. (1996). The Birds of North America.. Vol. 6. Academy of Natural Sciences & American Ornithologists’ Union, Philadelphia & Washington, D.C.
  - Dor, R., Carling, M.D., Lovette, I.J., Sheldon, F.H. and Winkler, D.W. (2012). Species trees for the tree swallows (Genus Tachycineta): an alternative phylogenetic hypothesis to the mitochondrial gene tree. Mol. Phylogenet. Evol. 65(1): 317–322.
  - Downing, C. (2005). New distributional information for some Colombian birds, with a new species for South America. Cotinga. 24: 13–15. <http://www.neotropicalbirdclub.org/pages/journal.asp?IssueID=24&Article_Link=Downing>
  - Erskine, A.J. (1979). Man’s influence on potential nesting sites and populations of swallows in Canada. Canadian Field-Naturalist. 93(4): 371–377.
  - Eynon, A.E. (1936). Rough-winged Swallow breeding in Rhode Island. Auk. 53(1): 83–84.
  - Finch, D. M. (1991). Population Ecology, Habitat Requirements, and Conservation of Neotropical Migratory Birds. General Technical Report RM-205. Rocky Mountain Forest and Range Experiment Station, Fort Collins, CO, USA.
  - Forcey, J.M. (2002). Notes on the birds of central Oaxaca, part III: Hirundinidae to Fringillidae. Huitzil 3(2): 43–55.
  - Gillespie, J.A. (1934). The homing instinct in the Rough-winged Swallow. Bird-Banding. 5: 43–44.
  - Godfrey, W. E. (1986). The Birds of Canada. Revised edition. National Museum of Natural Sciences, National Museums of Canada, Ottawa, Canada.
  - Griscom, L. (1929). Notes on the Rough-winged Swallow [Stelgidopteryx serripennis (Aud)] and its allies. Proc. New England Zool. Club 11: 67–72.
  - Griscom, L. (1932). The Distribution of Bird-Life in Guatemala. Bulletin of the American Museum of Natural History 64, New York. 439 pp.
  - Gómez de Silva, H. (2002). New distributional and temporal records of Mexican birds. Cotinga. 18: 89–92. <http://www.neotropicalbirdclub.org/wp-content/uploads/2016/05/Cotinga-18-2002-89-92.pdf>
  - Hellmayr, C. E. (1935). Catalogue of birds of the Americas. Volume 13. Part VIII. Field Museum of Natural History, Chicago, IL, USA. <https://www.biodiversitylibrary.org/page/2770994>
  - Hespenheide, H.A. (1975). Selective predation by two swifts and a swallow in Central America. Ibis. 117(1): 82–99.
  - Hill, J.R. (1988). Nest-depth preference in pipe-nesting Northern Rough-winged Swallows. J. Field Orn.. 59(4): 334–336.
  - Hoag, D.J. (2000). An instance of helping behavior in Northern Rough-winged Swallows. Wilson Bull.. 112(2): 281–282.
  - Howell, S. N. G., and S. Webb (1995). A Guide to the Birds of Mexico and Northern Central America. Oxford University Press, New York, NY, USA.
  - Husmann, K.H. (1981). Rough-winged Swallows nest in building. South Dakota Bird Notes 33(3): 59.
  - Jackson, J.A. (1993). Northern Rough-winged Swallows excavating at holes among the roots of upturned trees. Mississippi Kite 23: 13–14.
  - Johnsgard, P.A. (1979). Birds of the Great Plains. Breeding Species and Distribution. University of Nebraska Press, Lincoln, Nebraska & London.
  - Johnson, N.K. (1994). Old-school taxonomy versus modern biosystematics: species-level decisions in Stelgidopteryx and Empidonax. Auk. 111(3): 773–780.
  - Kaufman, K. (1996). Lives of North American Birds. Houghton Mifflin Company, Boston & New York.
  - Kaufman, K. (1996). Lives of North American Birds. Houghton Mifflin Company, Boston & New York.
  - Kingery, H.E. and Kingery, U.C. (1995). Gopher snake as predator at Long-billed Curlew and Rough-winged Swallow nests. J. Colo. Field Orn. 29(1): 18–19.
  - Lethaby, N. (1996). Identification of Tree, Northern Rough-winged, and Bank Swallows. Birding 28(2): 111–116.
  - Leukering, T. and Bradley, J. (1997). Some observations of birds on the central Mexican plateau. Western Birds. 28(3): 177–180. <https://sora.unm.edu/sites/default/files/journals/wb/v28n03/p0177-p0180.pdf>
  - Lunk, W.A. (1962). The Rough-winged Swallow Stelgidopterix ruficollis (Vieillot): a Study Based on its Breeding Biology in Michigan. Publications of the Nuttall Ornithological Club 4, Cambridge, Massachusetts. 155 pp.
  - MacMynowski, D.P., Root, T.L., Ballard, G. and Geupel, G.R. (2007). Changes in spring arrival of Nearctic-Neotropical migrants attributed to multiscalar climate. Global Change Biol. 13(11): 2239–2251. <http://onlinelibrary.wiley.com/doi/10.1111/j.1365-2486.2007.01448.x/abstract>
  - Michael, J.H. (1992). Intertidal nest of Northern Rough-winged Swallow. Wash. Birds 2: 23–24.
  - Moreno-Contreras, I., Mondaca, F., Robles-Morales, J., Jurado, M., Cruz, J., Alvidrez, A. and Robles-Carrillo, J. (2016). New distributional and temporal bird records from Chihuahua, Mexico. Bull. Brit. Orn. Club. 136(4): 272–286.
  - Navarro-Sigüenza, A.G. and Peterson, A.T. (2004). An alternative species taxonomy of the birds of Mexico. Biota Neotropica 4(2): 1–32.
  - Nickell, W.P. (1949). A large nest of the Rough-winged Swallow. Wilson Bull.. 61(3): 188–189. <https://sora.unm.edu/sites/default/files/journals/wilson/v061n03/p0188-p0189.pdf>
  - Paynter, R. A. (1955). The ornithogeography of the Yucatán Peninsula. Bulletin of the Peabody Museum of Natural History, Yale University 9:1–347. <https://www.biodiversitylibrary.org/item/40612#page/7/mode/1up>
  - Peck, G.K. and James, R.D. (1987). Breeding Birds of Ontario: Nidiology and Distribution. Vol. 2. Passerines. Life Sciences Miscellaneous Publication. Royal Ontario Museum, Toronto, Canada.
  - Peterjohn, B.G. (1989). The Birds of Ohio. Indiana University Press, Bloomington, Indiana.
  - Peterjohn, B.G., Sauer, J.R. and Robbins, C.S. (1995). Population trends from the North American Breeding Bird Survey. Pp. 3–39 in: Martin & Finch (1995).
  - Platania, S.P. and Clark, M.K. (1981). Rough-winged Swallow nesting in coastal North Carolina. Chat. 45(4): 100–102.
  - Price, J., Droege, S. and Price, A. (1995). The Summer Atlas of North American Birds. Academic Press, London.
  - Price, J., Droege, S. and Price, A. (1995). The Summer Atlas of North American Birds. Academic Press, London.
  - Price, J., S. Droege, and A. Price (1995). The Summer Atlas of North American Birds. Academic Press, New York, NY, USA.
  - Rappole, J.H., Morton, E.S., Lovejoy, T.E. and Ruos, J.L. (1995). Nearctic Avian Migrants in the Neotropics. 2nd edition. Conservation and Research Center, National Zoological Park, Smithsonian Institution, Front Royal, Virginia.
  - Ricklefs, R.E. (1972). Latitudinal variation in breeding productivity of the Rough-winged Swallow. Auk. 89(4): 826–836.
  - Ridgely, R. S., and J. A. Gwynne (1989). A Guide to the Birds of Panama with Costa Rica, Nicaragua, and Honduras. Second edition. Princeton University Press, Princeton, NJ, USA.
  - Robertson, W.B. and Kushlan, J.A. (1974). The southern Florida avifauna. Pp. 414–452 in: Gleason, P.J. ed. (1974). Environments of South Florida: Present and Past. Miami Geological Society Memoir 2, Miami, Florida.
  - Rohwer, S., Hobson, K.A. and Yang, S. (2011). Stable isotopes (δD) reveal east–west differences in scheduling of molt and migration in Northern Rough-winged Swallows (Stelgidopteryx serripennis). Auk. 128(3): 522–530.
  - Root, T. (1988). Atlas of Wintering North American Birds. An Analysis of Christmas Bird Count Data. The University of Chicago Press, Chicago, IL, USA.
  - Sauer, J.R. and Droege, S. (1992). Geographic patterns in population trends of Neotropical migrants in North America. Pp. 26–42 in: Hagan & Johnston (1992).
  - Screws, D.G. (1991). Unusual behavior in Northern Rough-winged Swallows. Oriole. 56: 44–45.
  - Sealy, S.G. (1982). Rough-winged Swallow scavenging adult midges. Wilson Bull.. 94(3): 368–369. <https://sora.unm.edu/sites/default/files/journals/wilson/v094n03/p0368-p0369.pdf>
  - Skutch, A. F. (1960). Life Histories of Central American Birds. Part 2. Families Vireonidae, Sylviidae, Turdidae, Troglodytidae, Paridae, Corvidae, Hirundinidae and Tyrannidae. Pacific Coast Avifauna 34. Cooper Ornithological Society, Berkeley, CA, USA.
  - Skutch, A.F. (1981). New Studies of Tropical American Birds. Publications of the Nuttall Ornithological Club 19. Cambridge, Massachusetts. 281 pp.
  - Small, A. (1994). California Birds: Their Status and Distribution. Ibis Publishing Company, Vista, California, USA.
  - Small, A. (1994). California Birds: their Status and Distribution. Ibis Publishing Company, Vista, California.
  - Stevenson, H.M. and Anderson, B.H. (1994). The Birdlife of Florida. University Press of Florida, Gainesville, Florida.
  - Stiles, F. G., and A. F. Skutch (1989). A Guide to the Birds of Costa Rica. Christopher Helm, London, UK.
  - Stiles, F.G. (1981). The taxonomy of Rough-winged Swallows (Stelgidopteryx; Hirundinidae) in southern Central America. Auk. 98(2): 282–293. <https://sora.unm.edu/sites/default/files/journals/auk/v098n02/p0282-p0293.pdf>
  - Stotz, D. F., J. W. Fitzpatrick, T. A. Parker, and D. K. Moskovits (1996). Neotropical Birds: Ecology and Conservation. University of Chicago Press, Chicago, Illinois, USA.
  - Van Fleet, W. (1876). Notes on the Rough-winged Swallow (Hirundo serripennis), in Pennsylvania. Bull. Nuttall Orn. Club 1: 9–11.
  - Wanless, R.M., Aguirre-Muñoz, A., Angel, A., Jacobsen, J.K., Keitt, B.S. and McCann, J. (2009). Birds of Clarion Island, Revillagigedo archipelago, Mexico. Wilson J. Orn.. 121(4): 745–751.
  - Weydemeyer, W. (1933). Nesting of the Rough-winged Swallow in Montana. Auk. 50(3): 362–363. <https://sora.unm.edu/sites/default/files/journals/auk/v050n03/p0362-p0363.pdf>
  - Winkler, K. (2006). Roosts and migrations of swallows. El Hornero. 21(2): 85–97.
  - Wolinski, R.A. (1980). Rough-winged Swallow feeding on fly larvae. Wilson Bull.. 92(1): 121–122. <https://sora.unm.edu/sites/default/files/journals/wilson/v092n01/p0121-p0122.pdf>
  - Yuri, T. and Rohwer, S. (1997). Molt and migration in the Northern Rough-winged Swallow. Auk. 114(2): 249–262. <https://sora.unm.edu/sites/default/files/journals/auk/v114n02/p0249-p0262.pdf>
